# Cost–utility analysis of normothermic and hypothermic *ex-situ* machine perfusion in liver transplantation

**DOI:** 10.1093/bjs/znab431

**Published:** 2021-12-14

**Authors:** Julia Zimmermann, Alexander W. Carter

**Affiliations:** Department of Health Policy, London School of Economics & Political Science, London, UK; London School of Hygiene and Tropical Medicine, London, UK; Addenbrooke's Transplant Unit, Cambridge University Hospitals NHS Foundation Trust, Cambridge, UK; Department of Health Policy, London School of Economics & Political Science, London, UK

*Dear Editor*

In recent years there has been renewed interest in *ex-situ* machine perfusion as a liver-preservation technique that promises better transplant outcomes and the potential to expand the donor pool safely to include older and sicker donors^1^^,^^2^. This evaluation adds to the literature as it compares relevant competitor devices and draws on effectiveness data from systematic review. The objective of this evaluation is to determine the cost–utility of *ex-situ* machine perfusion systems in elective adult liver transplant from the perspective of the National Health Service (NHS) in the UK.

The evaluation follows the National Institute of Health and Care Excellence’s reference case^3^. It uses a cohort model with a lifetime time horizon. The willingness-to-pay threshold is €23 650–€35 475 (£20 000–£30 000) per quality-adjusted life year (QALY). A Markov model conceptualizes the costs and QALYs for current practice and two comparator devices performing normothermic or hypothermic perfusion. The authors calculated base case incremental cost-effectiveness ratios (ICERs) and conducted extensive deterministic and probabilistic sensitivity analyses. They also calculated the expected value of perfect information. Full methods, a diagram of the model and the CHEERS statement are available in *Appendix 1*.

In the base case analysis, the ICER of both interventions was above the willingness-to-pay threshold, with an ICER of €241 300.07 (£204 059.25) per QALY and €1 288 668.47 (£1 089 783.06) per QALY for the Liver Assist™ (Organ Assist, Groningen, The Netherlands), performing hypothermic perfusion, and Metra™ (OrganOX, Oxford, UK), performing normothermic perfusion, respectively. Results of the base case and deterministic sensitivity analysis are shown in *Appendix 1*. In the deterministic sensitivity analysis neither interventions achieved cost-effectiveness at the willingness-to-pay threshold of €23 650–€35 475 (£20 000–£30 000) per QALY in any of the scenarios, but the interventions were dominated by current practice when organ utilization using the intervention was decreased. The variables that most influenced the ICER were the relative risk of early allograft dysfunction, the probability of organ utilization and the data source for the long-term outcomes.

The average results of the probabilistic sensitivity analysis are shown in *Fig. 1a*. On average, the Metra™ is dominated and offers fewer QALYs for higher costs. The Liver Assist™ offers lower costs for fewer QALYs with an ICER of €19 597.21 (£16 572.69) per QALY compared with current practice. The cost-effectiveness acceptability curve is shown in *Fig. 1b*. At a willingness-to-pay threshold of €23 650 (£20 000) per QALY, current practice has a probability of cost-effectiveness of 65 per cent, the Metra™ 3 per cent and the Liver Assist™ 32 per cent. The expected value of perfect information for different willingness-to-pay thresholds is also shown in *Fig. 1b*. At a willingness-to-pay threshold of €23 650 (£20 000) the expected value of perfect information is €14 192 734.74 (£12 002 312.68) per patient^4^.

**Fig. 1:**
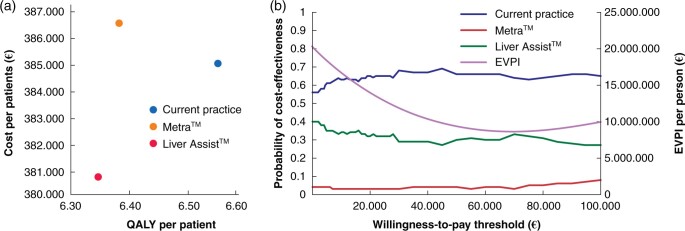
Results of the probabilistic sensitivity analysis **a** Mean results of the probabilistic sensitivity analysis. **b** Cost-effectiveness acceptability curve and expected value of perfect information (EVPI) at different willingness-to-pay thresholds.

This model is limited by the availability of data inputs. The authors were unable to assess heterogeneity as outcomes for livers from different types of donors are unclear. A recent economic evaluation also used a UK setting to evaluate the Metra™ against current practice and found the device to be cost-effective at a base case ICER of £7876 per QALY^5^. Important differences include that this evaluation was based on the outcomes of one trial only^6^. In particular the organ utilization rate found by this trial has a high risk of bias *Appendix 1*. Currently, *ex-situ* machine perfusion systems do not demonstrate cost-effectiveness to the NHS. However, the authors’ findings demonstrate much decision uncertainty. It is very likely that the opportunity cost of making a premature decision exceeds the cost of further research to reduce uncertainty. Therefore, cost-effectiveness should be re-evaluated when more information on organ utilization, long-term outcomes and costs are available.

*Disclosure*. The authors declare no conflicts of interest.

## Supplementary material

Supplementary material is available at *BJS* online.

## Supplementary Material

znab431_Supplementary_Data
