# Supplementary material for: Cost–utility analysis of normothermic and hypothermic ex-situ machine perfusion in liver transplantation
Source: Br J Surg. 2021 Dec 14;109(2):e31–2. doi: 10.1093/bjs/znab431 (PMC10364715; doi:10.1093/bjs/znab431)
Supplement: znab431_Supplementary_Data [file znab431_supplementary_data.docx]

# Supplementary material

# 1. Methods

This cost-utility analysis uses a Markov model and considers both costs and effects using an incremental analysis^1^. It follows CHEERS guidelines^2^ (end of document) and is guided by the NICE reference case^3^. Utility was measured in quality adjusted life years (QALY).

The setting of the evaluation is the UK. The perspective of the NHS in England was chosen as 6 of 7 liver transplant centres in the UK are located in England. The perspective includes all costs to the NHS and Personal Social Services in England^3^.

The target population was all patients on the adult elective deceased donor transplant list, reflecting the patient population in clinical trials^4^. Having gained the CE mark, the Metra™ and Liver Assist™, performing normothermic and hypothermic ex-situ machine perfusion respectively, were compared against current practice. Other machine perfusion devices that have gained the CE mark were not included as trial outcomes were not published in the peer-reviewed literature at the time of writing. Current practice is organ preservation in static cold storage (SCS). Both devices are evaluated as stationary, although livers can be transported on the Metra but not the Liver Assist^5,6^.

This evaluation has a life-time time horizon to capture all relevant costs and outcomes^3^. Life expectancy in liver transplantation is reported by Barber (2011)^7^.

Costs and outcomes were discounted at 3.5%, in line with the NICE reference case^3^. Cost and utility outcomes for SCS and both the devices were compared to identify dominance^1^**.** The willingness to pay threshold is 20,000-30,000 £/QALY, as set by NICE^3^.

**Model Structure**

The Markov model was built in Microsoft Excel (Microsoft Corporation, Redmond, WA). This model structure is suitable for capturing costs and effects over a life-time time horizon and is able to represent the cyclical nature of the waiting list and the long-term outcomes (35). The model was run with a cohort simulation using 432 average UK liver transplant patients and a cycle length of one month. Average patient characteristics were determined using NHSBT data^8^. This cycle length was chosen as mean hospital admissions for a liver transplant are 24.8 days long^9^. Liver transplant consultants at Cambridge University Hospitals NHS Foundation Trust (CUH) were consulted to ensure that model structure, assumptions and choice of parameters were an appropriate reflection of clinical practice and current knowledge.

Model structure is shown in Supplementary material 1 Figure 1. Patients started in the “Waiting List” health state from which they could either receive a transplant or die, reflecting waiting list mortality^8^. Death was defined as an absorbing state^1^. If patients received a transplant, they would either have no complications, a biliary complication, Early Allograft Dysfunction (EAD) or Primary Non-Function (PNF). While there is no consensus definition in the literature for many of these complications, this analysis is guided by definitions used in a recent meta-analysis^4^. Thereby, biliary complications are defined as all complications related to the biliary duct^4^. EAD is defined according to Olthoff (2010)^10^. PNF is defined as irreversible graft dysfunction that requires an emergency liver transplant during the first 10 days post-operatively^4,11^.


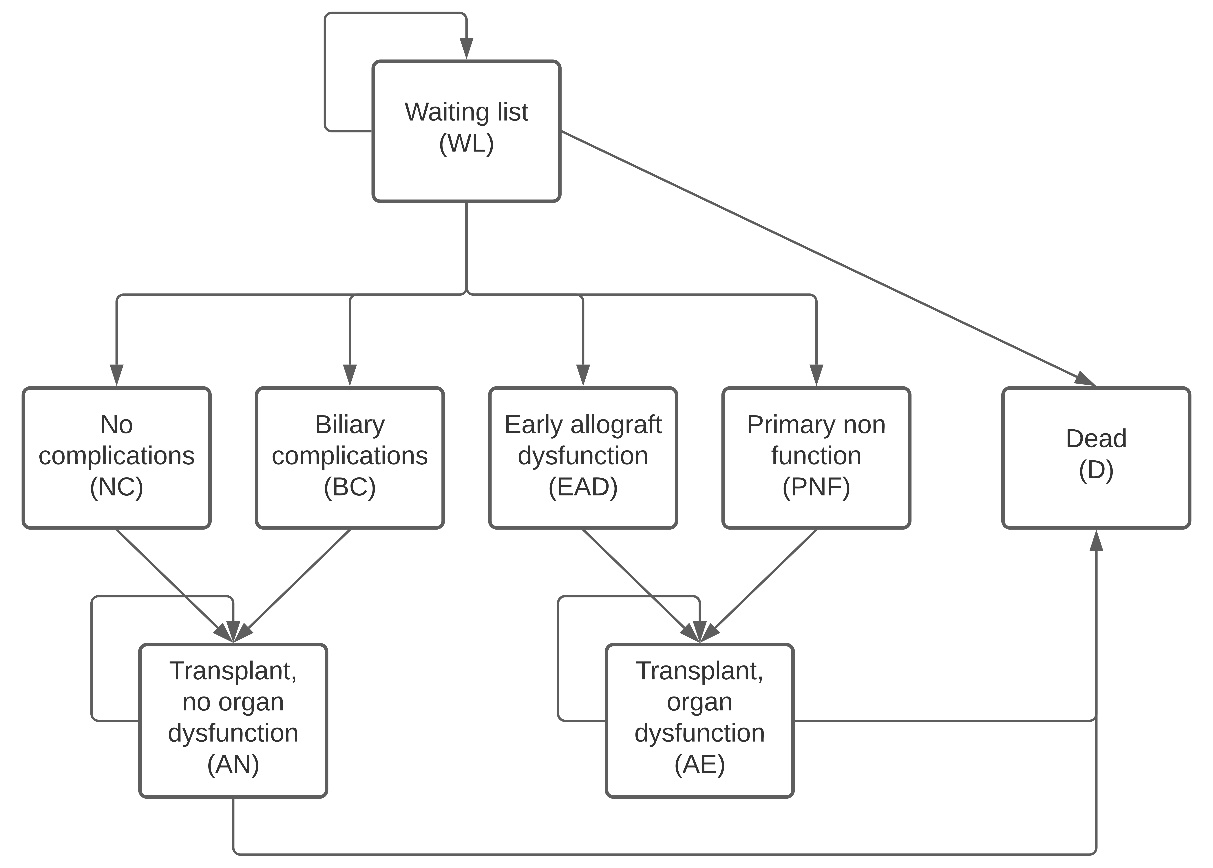


Supplementary material 1 Figure 1. Schematic of the model structure.

Health states in which patients received a transplant were tunnel states. This is a simplification, as peri-transplant mortality occurs occasionally and is known to be high in PNF^12^. As the probability of PNF is low, mortality caused by it was deemed negligible and unlikely to change results.

The model differentiates between long-term outcomes for organs with good function and poor function. Resolved biliary complications have the same long-term outcomes as uncomplicated liver transplants^13^. Outcomes of livers with EAD are significantly different to livers without EAD, both in the short and the long-term^10,14^. Patients with PNF also have significantly worse outcomes^15^. It is well known that the first year post transplant has both higher risks of complications and graft failure than subsequent years^8^. In addition, transplant outcomes for re-transplantation are known to be worse than outcomes for the first graft^16^. It was deemed important to differentiate between good and poor long-term outcomes. However, the change in outcomes over time and the probability of requiring a re-transplant were so small that it was assumed that these would not affect overall outcomes.

Another assumption of the model is the effect of machine perfusion on the proportion of donated organs that are suitable for transplant. This determines the annual volume of transplants. Machine perfusion devices have the potential to increase organ use^17,18^. It is also possible that organ use declines in response to implementing the device, as this introduces further logistical and capacity challenges which may prevent the transplant from proceeding. In the base case scenario, we assumed no difference in organ utilisation between current practice and machine perfusion. We assumed that the probability of receiving a transplant was relative to the proportion of organs that can be transplanted, or rate of organ utilisation. We explored this uncertainty in the deterministic and probabilistic sensitivity analysis (PSA).

**Model Parameters**

Parameters and their sources are displayed in Supplementary material 1 Table 1. The device cost and an extra cost which accounts for running the device on discarded organs were added to every transplant, including re-transplants. To calculate post-transplant costs for both “alive with transplant” states, we assumed that graft failure would result in re-transplantation in all cases.

Supplementary material 1 Table 1. Model input parameters, their source and the distribution used for PSA.

|  | **Definition** | **Value** | **Distribution** | **Mean**  **(* Number of events)** | **Standard error**  **(* Number of non-events)** | **Source** |
| --- | --- | --- | --- | --- | --- | --- |
| **Costs** | |  |  |  |  |  |
|  | Cost waiting list | £1 312.75 | Gamma | £1 312.75 | £1 312.75 | Tanajewski et al^19^ |
|  | Cost transplant, no complications | £17 552.33 | Fixed |  |  | NHS reference costs 2018/19^20^ (GA15A "elective") |
|  |  |  |  |  |  |  |
|  |  |  |  |  |  |  |
|  | Cost transplant with Biliary complications | £21 435.19 | Gamma | £21 435.19 | £21 435.19 | Calculation described in text, Englesbe et al.^21^ |
|  |  |  |  |  |  |  |
|  |  |  |  |  |  |  |
|  | Cost transplant with EAD | £20 731.32 | Gamma | £20 731.32 | £20 731.32 | NHS reference costs 2018/19^20^ (GA15A "long stay") as known longer stay ^22^ |
|  |  |  |  |  |  |  |
|  |  |  |  |  |  |  |
|  |  |  |  |  |  |  |
|  | Base cost alive with transplant | £2 909.17 | Gamma | £2 909.17 | £2 909.17 | Tanajewski et al.^19^ |
|  | Cost 2nd transplant / PNF | £32 120.76 | Gamma | £32,120.76 | £32,120.76 | Calculated described in text using Azoulay et al.^23^ |
|  |  |  |  |  |  |  |
|  |  |  |  |  |  |  |
|  | Probability of a 2nd liver transplant when in state "AN" | 0.0058 | Fixed |  |  | Olthoff et al.^10^ |
|  |  |  |  |  |  |  |
|  | Probability of a 2nd liver transplant when in state "AE" | 0.0435 | Fixed |  |  | Olthoff et al.^10^ |
|  |  |  |  |  |  |  |
|  | Extra cost for Metra™ discarded organs | £1 277.05 |  |  |  | Calculated |
|  | Extra cost for Liver Assist™ discarded organs | £784.64 |  |  |  | Calculated |
|  | Cost Metra™ per transplant | £7 337.21 | Fixed |  |  | See Supplementary material 3 |
|  | Cost Liver Assist™ per transplant | £4 508.11 | Fixed |  |  | See Supplementary material 3 |
| **Utility** | |  |  |  |  |  |
|  | HRQoL Waiting list | 0.53 | Beta | 0.53 | 0.53 | Ratcliffe et al.^24^ |
|  | HRQoL NC | 0.64 | Beta | 0.64 | 0.64 | Ratcliffe et al.^24^ |
|  | HRQoL BC | 0.64 | Beta | 0.64 | 0.34 | Ratcliffe et al.^24^ |
|  | HRQoL EAD | 0.64 | Beta | 0.64 | 0.64 | Ratcliffe et al.^24^ |
|  | HRQoL PNF | 0.64 | Beta | 0.64 | 0.64 | Ratcliffe et al.^24^ |
|  | HRQoL state “AN” | 0.76 | Beta | 0.76 | 0.015 | Ratcliffe et al.^24^ |
|  | HRQoL state “AE” | 0.76 | Beta | 0.76 | 0.015 | Ratcliffe et al.^24^ |
| **Transition probabilities (TP)** | |  |  |  |  |  |
|  | TP WL to WL | 0.9191 |  |  |  | Calculated |
|  | % organ utilisation current practice | 0.8518 | Fixed |  |  | NHSBT^25^ |
|  | TP WL to a transplant | 0.0628 | Fixed |  |  | NHSBT^25^ |
|  |  |  |  |  |  |  |
|  | TP WL to NC | 0.9749 |  |  |  | Calculated |
|  | TP WL to BC | 0.0077 | Fixed |  |  | Nasralla et al.^11^ |
|  | TP WL to EAD | 0.017 | Fixed |  |  | Nasralla et al.^11^ |
|  | TP WL to PNF | 0.0003 | Fixed |  |  | Al-Freah et al.^12^ |
|  | TP WL to D | 0.0181 | Fixed |  |  | NHSBT ^25^ |
|  | TP NC to AN | 1 | Fixed |  |  | Tunnel state |
|  | TP BC to AN | 1 | Fixed |  |  | Tunnel state |
|  | TP EAD to AE | 1 | Fixed |  |  | Tunnel state |
|  | TP PNF to AE | 1 | Fixed |  |  | Tunnel state |
|  | TP AN to AN | 0.997 |  |  |  | Calculated |
|  | TP AN to D | 0.003 | Fixed |  |  | Olthoff et al.^10^ |
|  | TP AE to AE | 0.9659 |  |  |  | Calculated |
|  | TP AE to D | 0.0341 | Fixed |  |  | Olthoff et al.^10^ |
|  | % Organ utilisation Metra™ | 0.8518 | Beta | 1 065* | 48* | NHSBT ^25^ , Mergental et al.^17^ |
|  | TP WL to a transplant, Metra™ | 0.0628 |  |  |  | Calculated, NHSBT^25^ |
|  |  |  |  |  |  |  |
|  | RR Biliary complications Metra™ | 0.7 | Log-normal | 0.70 | 4.17 | Jia et al.^4^ |
|  | RR EAD Metra™ | 0.44 | Log-normal | 0.44 | 2.71 | Jia et al.^4^ |
|  | RR PNF Metra™ | 0.6 | Log-normal | 0.60 | 17.52 | Jia et al.^4^ |
|  | % Organ utilisation Liver Assist™ | 0.8518 | Beta | 1 065* | 48* | NHSBT^25^ , assumed similar performance to Metra™ based on Rayar et al.^18^ |
|  | TP WL to a transplant, Liver Assist™ | 0.0628 |  |  |  | Calculated, NHSBT^25^ |
|  |  |  |  |  |  |  |
|  | RR Biliary complications Liver Assist™ | 0.45 | Log-normal | 0.45 | 3.51 | Jia et al.^4^ |
|  | RR EAD Liver Assist™ | 0.49 | Log-normal | 0.49 | 3.18 | Jia et al.^4^ |
|  | RR PNF Liver Assist™ | 0.60 | Log-normal | 0.60 | 17.52 | Jia et al.^4^ |
|  |  |  |  |  |  |  |
|  | Discount rate costs | 3.50% | Fixed |  |  | NICE^3^ |
|  | Discount rate utility | 3.50% | Fixed |  |  | NICE^3^ |

**Transition Probabilities**

Rates and risks in the literature were transformed into monthly transition probabilities^26^. Complications were assumed to be rare. Therefore, Odds Ratios were assumed to be identical to other measures of RR^27^.

The probabilities of death and graft failure, in the long-term, were taken from Olthoff^10^. Alternate findings reported by Lee (2016) are explored in the sensitivity analysis, but were deemed less appropriate as their patient population included super-urgent transplant patients^14^.

There are two recent meta-analyses of machine perfusion in liver transplant^4,28^. The more recent analysis contains all studies of the first meta-analysis^4,28^. Both excluded sub-hypothermic or graduated temperature protocols or trials reporting duplicate data and used a fixed-effects model as they found little evidence of heterogeneity^4,28^.

Both meta-analyses summarised trials that used normothermic and hypothermic machine perfusion respectively. As most trials with hypothermic machine perfusion were conducted on the Liver Assist™ and most trials with normothermic machine perfusion with the Metra™ we assumed that the outcome data for hypothermic machine perfusion can be attributed to the Liver Assist™ and the outcome data for normothermic machine perfusion can be attributed to the Metra™. Some small studies included in the meta-analyses used other machine perfusion devices or did not follow this pattern of device allocation, but due to a paucity of comparable trials and similar results, this was deemed acceptable. Recently published trial results are in keeping with the results of the meta-analysis^29,30^.

Only 3 trials had reported cases of PNF^4^. None of these trials were adequately powered to assess the incidence of this rare complication. The meta-analysis by Jia did not differentiate between normothermic and hypothermic perfusion for PNF^4^. This is most likely to be representative of outcomes and we used this RR for both devices.

To ensure no relevant papers had been missed, a systematic literature search was conducted on the 05.07.2021 to identify relevant papers not included in the most recent meta-analysis. EMBASE, Pubmed/Medline and the Cochrane library were searched. Search results are shown in Supplementary material 1 Figure 2.

The search was conducted using the following search phrase, which was adapted from Jia 2020^4^ and Bellini 2019^28^:

((liver) OR (hepat*)) AND (trial) AND ((transplant) OR (graft) OR (donor) OR (donat*)) AND ((machine) OR (device)) AND ((hypotherm*) OR (normotherm*) OR (oxygen*)) NOT ((animal*) OR (porcine) OR (rat) OR ("not human"))

Inclusion criteria: English language papers published between November 2019 and July 2021. Papers must investigate adult, elective liver transplant. They must be a human, clinical study. The study design must be an observational study or a randomised controlled trial.

Exclusion criteria: Any trials investigating split livers or live donors. Any trial intervention that does not use the Metra™ or Liver Assist™ device for ex-vivo perfusion. Temperature protocols that fall outside the categories of normothermic or hypothermic perfusion.

Supplementary material 1 Figure 2: Outcomes of the systematic literature search.


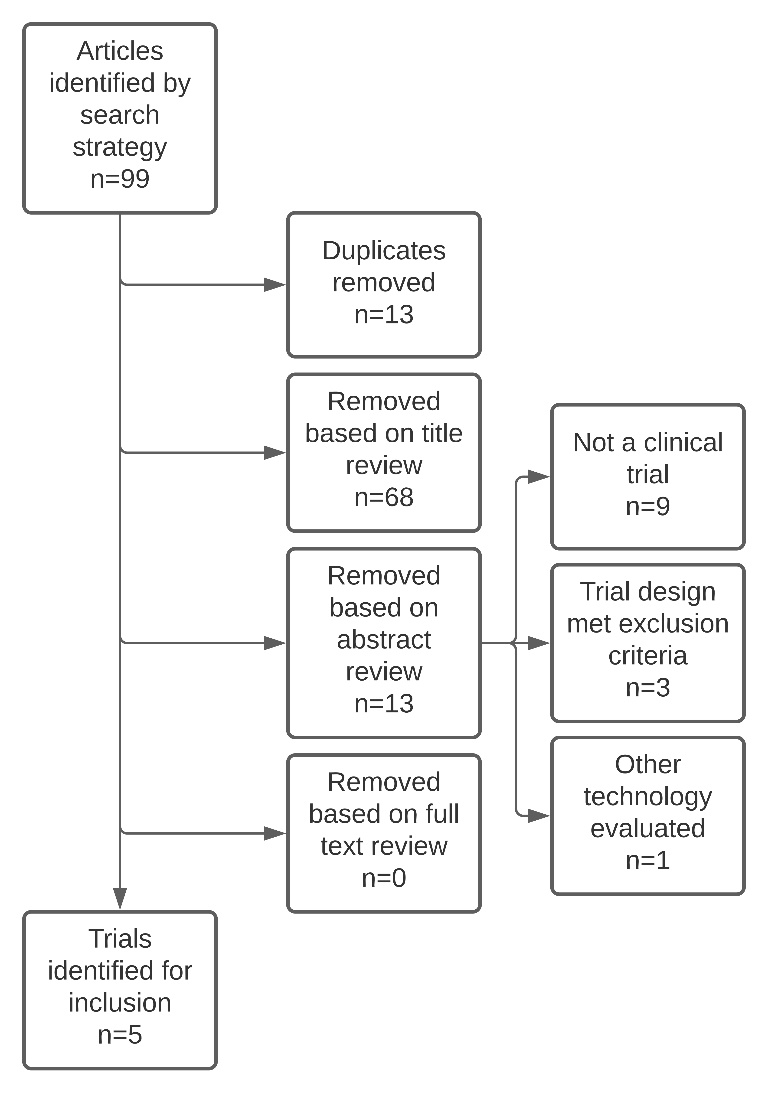


This identified five relevant trials^18,29–32^. Results of these trial were taken into consideration in the base case model and sensitivity analysis.

**Costs**

All costs were calculated in British Pound Sterling and inflated to 2018-19 prices. Currency conversions were made using purchasing power parities^33^. Hospital costs were inflated using the Health and Social Care Index provided by Personal Social Services Research Unit (PSSRU)^34^. Medication and device costs were inflated using the Retail Price Index^35^.

Liver Assist™ and Metra™ device costs were provided by the manufacturer (Supplementary material 1 Table 2 and 3).

Supplementary material 1 Table 2. Costing of the Metra™ device. Machine perfusion solutions were costed according to the protocol used by the liver transplant team at Cambridge University Hospitals NHS Foundation Trust (CUH).

| Metra | | Cost per transplant in GBP, (2018/19) | Source of price |
| --- | --- | --- | --- |
| **Variable costs** | |  |  |
|  | Disposable kit | £ 6 000.00 | Organox |
|  | Machine perfusion solutions | £ 538.85 | CUH, BNF^36^, NHS reference costs 2018/19^20^ |
|  | Laboratory tests | £ 196.00 | CUH, NHS reference costs 2018/19^20^ |
|  | Staff costs | £ 238.44 | PSSRU^34^ |
| **Capital costs** | |  |  |
|  | Perfusion room | £ 149.63 | CUH |
|  | Metra device | £ 214.29 | Organox |
|  | Total cost | £ 7,337.21 |  |

Supplementary material 1 Table 3. Costing of the Liver Assist™ device.

| Liver Assist | | Cost per transplant in GBP, 2018/19 | Source of price |
| --- | --- | --- | --- |
| **Variable costs** | |  |  |
|  | Disposable kit and shipping | £ 3 531.50 | Organ Assist |
|  | Machine perfusion solutions | £ 283.08 | Rayar et al^18^ |
|  | Staff cost | £ 443.48 | PSSRU^34^ |
| **Capital costs** | |  |  |
|  | Perfusion room | £ 149.63 | CUH |
|  | O2 blender | £ 0.95 | CUH |
|  | Liver Assist device including annual servicing cost | £ 99.47 | Organ Assist |
|  | Total cost | £ 4,508.10 |  |

The cost per procedure was calculated for all capital costs^37,38^. Costs for a dedicated perfusion room fed into the cost per procedure. The perfusion room costs assume a one-time cost covering the entire time horizon. This may be an underestimation. The Liver Assist™ requires a sterile environment to run, while the Metra™ can be moved to an unsterile environment once set up. This is explored in the sensitivity analysis. The costs of the Liver Assist™ device was calculated using the same methodology assuming a 7 year lifetime of the device^39^ with no resale value^1^. The cost of the Metra™ device is included in the annual service agreement. We assumed one device per transplant centre and an annual caseload per device of 140, the average annual caseload per UK transplant centre^8^.

Top-down costing was used for all other parameters. Waiting list and transplant costs were based on the costs of decompensated cirrhosis caused by NAFLD^19^. Although this does not reflect the cost of different aetiologies of chronic liver disease, costs for decompensated cirrhosis are often estimated to be similar^40^. The cost of organ retrieval and transport were assumed to be contained within the NHS reference costs for a liver transplant. Second transplants were estimated to be 1.83 times higher than first transplant costs^23^, as used in a previous economic evaluation^41^. The same method was used to estimate the cost of biliary complications; a US costing study found that transplants with biliary complications were 1.22 times more expensive than a transplant without complications^21^.

**Utility**

A UK based study by Ratcliffe (2002) ascertaining health related quality of life (HRQoL) in liver transplant patients was chosen to inform this evaluation^42^. Although there is an estimation from meta-analysis^43^, the Ratcliffe study follows best practice as the data originates from the population of interest^1,3^. Those who died were assumed to have a utility of 0, in keeping with the EQ-5D scale^1^. The effect of machine perfusion on health utility has not been researched to date.

**Exploring Uncertainty**

Both deterministic and probabilistic sensitivity analysis were performed to test the robustness of the model. Structural assumptions have been described in detail and structural uncertainty was not formally quantified^44^. Methodological uncertainty was explored in the one-way sensitivity analysis by exploring the alternate data for long-term outcomes^14^, varying discount rates according to the NICE reference case^3^ and by varying the time horizon^1^. Parameter uncertainty was addressed in the one-way and probabilistic sensitivity analysis^1^. For the one-way sensitivity analysis each parameter was varied individually. The 95% Confidence Interval (95% CI) was used to inform high and low estimates where available. Parameters were varied by +/- 10% if a 95% CI was not available.

We attempted to model heterogeneity due to different types of organ donor (DBD, DCD, extended criteria donor). Due to a paucity of data, both for the baseline values and RR, it was not possible to conduct the subgroup analysis.

PSA included uncertain stochastic parameters and was conducted using a Monte-Carlo simulation of 10,000 replications. Assigned distributions are reported in Table 1^26^. The method of moments was used to calculate alpha and beta from mean and SE where applicable^26^. The Standard Error (SE) for all costs was assumed to be the same as the value of the cost as no SE had been reported^26^. Organ utilisation, and therefore the probability of receiving a transplant, was varied using a binomial distribution. The liver allocation policy is a main determinant of waiting list mortality^45^. This variable was deemed independent of the probability of receiving a transplant and was not varied. Therefore, patients either received a transplant or remained part of the waiting list, which is a binary outcome.

Net monetary benefit was calculated for willingness-to-pay thresholds up to £100,000 and this was used to plot the cost-effectiveness acceptability curve. The expected value of perfect information (EVPI) per person for a willingness to pay threshold of £20,000 was calculated^26^**.**

This study did not require ethical approval (London School of Hygiene and Tropical Medicine REF 21961).

Acknowledgements

The authors would like to thank to the transplant department at Cambridge University Hospitals NHS Foundation Trust. Particularly, we would like to thank Professor Christopher Watson for his expert insight into national liver transplant services.

Funding: None to declare.

Competing interests: None to declare.

Bibliography

1 Drummond MF, Sculpher MJ, Claxton K, Stoddart G, Torrance GW. Methods for the economic evaluation of health care programmes. Drummond M, Sculpher MJ, Claxton K, Stoddart GL, Torrance GW, editors. Methods Econ. Eval. Heal. care Program. OUP Oxford; 2015.

2 Husereau D, Drummond M, Petrou S, Carswell C, Moher D, Greenberg D, *et al.* Consolidated Health Economic Evaluation Reporting Standards (CHEERS) statement. *BMJ* [Internet]. 2013 [cited 2020 Jan 12]; **346**: f1049. Available from: https://www.bmj.com/content/bmj/346/bmj.f1049.full.pdf

3 National Institute for Health and Care Excellence (NICE). The reference case | Guide to the methods of technology appraisal 2013 | Guidance | NICE [Internet]. [cited 2020 Aug 16]. Available from: https://www.nice.org.uk/process/pmg9/chapter/the-reference-case

4 Jia J, Zheng S. A Systematic Review and Meta-Analysis of Machine Perfusion vs . Static Cold Storage of Liver Allografts on Liver Transplantation Outcomes : The Future Direction of Graft Preservation. 2020; **7**.

5 Ceresa CDL, Nasralla D, Watson CJE, Butler AJ, Coussios CC, Crick K, *et al.* Transient Cold Storage Prior to Normothermic Liver Perfusion May Facilitate Adoption of a Novel Technology. *Liver Transplant Off Publ Am Assoc Study Liver Dis Int Liver Transplant Soc*. United States; 2019 Oct; **25**: 1503–1513.

6 Bral M, Dajani K, Leon Izquierdo D, Bigam D, Kneteman N, Ceresa CDL, *et al.* A Back-to-Base Experience of Human Normothermic Ex Situ Liver Perfusion: Does the Chill Kill? *Liver Transplant*. 2019; **25**: 848–858.

7 Barber K, Blackwell J, Collett D, Neuberger J. Life expectancy of adult liver allograft recipients in the UK. [cited 2020 Nov 8]; Available from: www.gutjnl.com

8 NHS Blood and Transplant. ANNUAL REPORT ON LIVER TRANSPLANTATION [Internet]. 2019. Available from: https://nhsbtdbe.blob.core.windows.net/umbraco-assets-corp/16782/nhsbt-liver-transplantation-annual-report-2018-19.pdf

9 Tovikkai C, Charman SC, Praseedom RK, Gimson AE, Meulen J van der. Time spent in hospital after liver transplantation: Effects of primary liver disease and comorbidity. *World J Transplant* [Internet]. Baishideng Publishing Group Inc.; 2016 [cited 2020 Aug 16]; **6**: 743. Available from: /pmc/articles/PMC5175234/?report=abstract

10 Olthoff KM, Kulik L, Samstein B, Kaminski M, Abecassis M, Emond J, *et al.* Validation of a Current Definition of Early Allograft Dysfunction in Liver Transplant Recipients and Analysis of Risk Factors. *Liver Transplant*. 2010; **16**: 943–949.

11 Nasralla D, Coussios CC, Mergental H, Zeeshan Akhtar M, Butler AJ, Ceresa DL, *et al.* A randomized trial of normothermic preservation in liver transplantation. *Nature* [Internet]. 2018 [cited 2020 Apr 9]; Available from: https://doi.org/10.1038/s41586-018-0047-9

12 Al-Freah MAB, McPhail MJW, Dionigi E, Foxton MR, Auzinger G, Rela M, *et al.* Improving the Diagnostic Criteria for Primary Liver Graft Nonfunction in Adults Utilizing Standard and Transportable Laboratory Parameters: An Outcome-Based Analysis. *Am J Transplant*. 2017; **17**: 1255–1266.

13 Ogiso S, Kamei H, Onishi Y, Kurata N, Jobara K, Kawashima H, *et al.* Decreased long-term graft survival in persistent biliary complications after right-lobe living-donor liver transplantation. *Clin Transplant*. 2019; **34**: 1–7.

14 Lee DD, Croome KP, Shalev JA, Musto KR, Sharma M, Keaveny AP, *et al.* Early allograft dysfunction after liver transplantation: An intermediate outcome measure for targeted improvements. *Ann Hepatol* [Internet]. Elsevier; 2016; **15**: 53–60. Available from: http://dx.doi.org/10.5604/16652681.1184212

15 Kemmer N, Secic M, Zacharias V, Kaiser T, Neff GW. Long-term Analysis of Primary Nonfunction in Liver Transplant Recipients. *Transplant Proc*. 2007; **39**: 1477–1480.

16 Marudanayagam R, Shanmugam V, Sandhu B, Gunson BK, Mirza DF, Mayer D, *et al.* Liver retransplantation in adults: A single-centre, 25-year experience. *HPB* [Internet]. John Wiley and Sons Inc; 2010 [cited 2020 Jul 10]; **12**: 217–224. Available from: https://pubmed.ncbi.nlm.nih.gov/20590890/

17 Mergental H, Laing RW, Kirkham AJ, Perera MTPR, Boteon YL, Attard J, *et al.* Transplantation of discarded livers following viability testing with normothermic machine perfusion. *Nat Commun* [Internet]. Springer US; 2020; **11**. Available from: http://dx.doi.org/10.1038/s41467-020-16251-3

18 Rayar M, Beaurepaire JM, Bajeux E, Hamonic S, Renard T, Locher C, *et al.* Hypothermic Oxygenated Perfusion Improves Extended Criteria Donor Liver Graft Function and Reduces Duration of Hospitalization Without Extra Cost: The PERPHO Study. *Liver Transplant*. 2021; **27**: 349–362.

19 Tanajewski L, Harris R, Harman DJ, Aithal GP, Card TR, Gkountouras G, *et al.* Economic evaluation of a community-based diagnostic pathway to stratify adults for non-alcoholic fatty liver disease: A Markov model informed by a feasibility study. *BMJ Open*. 2017; **7**: 1–11.

20 Improvement N. Reference costs | NHS Improvement [Internet]. 2018 [cited 2019 Oct 31]. Available from: https://improvement.nhs.uk/resources/reference-costs/

21 Englesbe MJ, Dimick J, Mathur A, Ads Y, Welling TH, Pelletier SJ, *et al.* Who pays for biliary complications following liver transplant? A business case for quality improvement. *Am J Transplant*. 2006; **6**: 2978–2982.

22 Croome KP, Hernandez-Alejandro R, Chandok N. Early allograft dysfunction is associated with excess resource utilization after liver transplantation. *Transplant Proc* [Internet]. Elsevier Inc.; 2013; **45**: 259–264. Available from: http://dx.doi.org/10.1016/j.transproceed.2012.07.147

23 Azoulay D, Linhares MM, Huguet E, Delvart V, Castaing D, Adam R, *et al.* Decision for retransplantation of the liver: An experience- and cost-based analysis. *Ann Surg* [Internet]. Lippincott, Williams, and Wilkins; 2002 Dec 1 [cited 2020 Jul 15]; **236**: 713–721. Available from: /pmc/articles/PMC1422637/?report=abstract

24 Bryan S, Ratcliffe J, Neuberger JM, Burroughs AK, Gunson BK, Buxton MJ. Health-related quality of life following liver transplantation. *Qual Life Res*. 1998; **7**: 115–120.

25 NHS Blood and Transplant. Organ Donation and Transplantation - Activity Report 2018-2019 [Internet]. 2019. Available from: http://www.odt.nhs.uk.

26 Briggs A, Sculpher MJ, Claxton K. Decision Modelling for Health Economic Evaluation - Andrew Briggs, Mark Sculpher, Karl Claxton - Oxford University Press [Internet]. Oxford: Oxford University Press; 2006 [cited 2020 Aug 16]. Available from: https://global.oup.com/academic/product/decision-modelling-for-health-economic-evaluation-9780198526629?cc=gb&lang=en&

27 Webb P, Bain C, Page A. Essential Epidemiology [Internet]. Essent. Epidemiol. Cambridge University Press; 2016 [cited 2020 Aug 16]. Available from: /core/books/essential-epidemiology/C44A43C69EC5198363B217F7ECF86C7A

28 Bellini MI, Nozdrin M, Yiu J, Papalois V. Machine Perfusion for Abdominal Organ Preservation: A Systematic Review of Kidney and Liver Human Grafts. *J Clin Med*. MDPI AG; 2019 Aug 15; **8**: 1221.

29 van Rijn R, Schurink IJ, de Vries Y, van den Berg AP, Cerisuelo MC, Murad SD, *et al.* Hypothermic machine perfusion in liver transplantation — A randomized trial. *N Engl J Med* [Internet]. 2021; **384**: 1391‐1401. Available from: https://www.cochranelibrary.com/central/doi/10.1002/central/CN-02259659/full

30 Ravaioli M, De Pace V, Angeletti A, Comai G, Vasuri F, Baldassarre M, *et al.* Author Correction: Hypothermic Oxygenated New Machine Perfusion System in Liver and Kidney Transplantation of Extended Criteria Donors: First Italian Clinical Trial. *Sci Rep* [Internet]. United Kingdom: NLM (Medline); 2020 Apr; **10**: 6063. Available from: http://ovidsp.ovid.com/ovidweb.cgi?T=JS&PAGE=reference&D=emexb&NEWS=N&AN=631469058

31 Mergental H, Laing RW, Kirkham AJ, Perera MTPR, Boteon YL, Attard J, *et al.* Transplantation of discarded livers following viability testing with normothermic machine perfusion. *Nat Commun*. 2020 Jun; **11**: 2939.

32 MacConmara M, Hanish SI, Hwang CS, De Gregorio L, Desai DM, Feizpour CA, *et al.* Making Every Liver Count: Increased Transplant Yield of Donor Livers Through Normothermic Machine Perfusion. *Ann Surg* [Internet]. United States: NLM (Medline); 2020; **272**: 397–401. Available from: http://ovidsp.ovid.com/ovidweb.cgi?T=JS&PAGE=reference&D=emexb&NEWS=N&AN=632440966

33 The World Bank. PPP conversion factor, GDP (LCU per international $) [Internet]. [cited 2020 Aug 16]. Available from: https://data.worldbank.org/indicator/PA.NUS.PPP

34 Curtis LA, Burns A. Unit costs of Health and Social Care 2019 [Internet]. 2019. Available from: https://doi.org/10.22024/UniKent%2F01.02.79286

35 Office for National Statistics. Retail Prices Index: Long run series: 1947 to 2019 [Internet]. [cited 2020 Aug 16]. Available from: https://www.ons.gov.uk/economy/inflationandpriceindices/timeseries/cdko/mm23

36 National Institute for Health and Care Excellence (NICE). BNF British National Formulary [Internet]. BNF. 2020 [cited 2020 Aug 23]. Available from: https://bnf.nice.org.uk/

37 Mihaljevic T, Koprivanac M, Kelava M, Goodman A, Jarrett C, Williams SJ, *et al.* Value of robotically assisted surgery for mitral valve disease. *JAMA Surg* [Internet]. American Medical Association; 2014 [cited 2020 Aug 16]; **149**: 679–686. Available from: /pmc/articles/PMC4262248/?report=abstract

38 Ramsay C, Pickard R, Robertson C, Close A, Vale L, Armstrong N, *et al.* Systematic review and economic modelling of the relative clinical benefit and cost-effectiveness of laparoscopic surgery and robotic surgery for removal of the prostate in men with localised prostate cancer. *Health Technol Assess*. 2012; **16**: 1–313.

39 Organ Assist. Liver Assist brochure [Internet]. [cited 2020 Aug 16]. Available from: https://www.organ-assist.nl/wp-content/uploads/2019/09/Liver-Assist-Brochure.pdf

40 Johansen P, Howard D, Bishop R, Moreno SI, Buchholtz K. Systematic Literature Review and Critical Appraisal of Health Economic Models Used in Cost-Effectiveness Analyses in Non-Alcoholic Steatohepatitis: Potential for Improvements. *Pharmacoeconomics* [Internet]. Springer International Publishing; 2020; **38**: 485–497. Available from: https://doi.org/10.1007/s40273-019-00881-7

41 Muduma G, Odeyemi I, Pollock RF. A cost-utility analysis of prolonged-release tacrolimus relative to immediate-release tacrolimus and ciclosporin in liver transplant recipients in the UK. *J Med Econ*. 2016; **19**: 995–1002.

42 Ratcliffe J, Longworth L, Young T, Bryan S, Burroughs A, Buxton M. Assessing health-related quality of life pre- and post-liver transplantation: A prospective multicenter study. *Liver Transplant*. 2002; **8**: 263–270.

43 McLernon DJ, Dillon J, Donnan PT. Health-state utilities in liver disease: A systematic review. *Med Decis Mak*. 2008; **28**: 582–592.

44 Briggs AH, Weinstein MC, Fenwick EAL, Karnon J, Sculpher MJ, Paltiel AD. Model parameter estimation and uncertainty: A report of the ISPOR-SMDM modeling good research practices task force-6. *Value Heal*. Elsevier; 2012 Sep 1; **15**: 835–842.

45 Robinson C. POLICY POL196 Deceased Donor Liver Distribution and Allocation [Internet]. 2019. Available from: https://www.odt.nhs.uk/transplantation/tools-policies-and-guidance/policies-and-guidance/

CHEERS statement

CHEERS checklist from ^2^.

| Section/item | Item No | Recommendation | Reported on page No/ line No |
| --- | --- | --- | --- |
| **Title and abstract** | | | |
| Title | 1 | Identify the study as an economic evaluation or use more specific terms such as “cost-effectiveness analysis”, and describe the interventions compared. | Title page |
| Abstract | 2 | Provide a structured summary of objectives, perspective, setting, methods (including study design and inputs), results (including base case and uncertainty analyses), and conclusions. | N/A for this type of article |
| **Introduction** | | | |
| Background and objectives | 3 | Provide an explicit statement of the broader context for the study. | Main text page 1, lines 1-3 |
|  |  | Present the study question and its relevance for health policy or practice decisions. | Main text page 1 lines 3-7 |
| **Methods** | | | |
| Target population and subgroups | 4 | Describe characteristics of the base case population and subgroups analysed, including why they were chosen. | Supplementary material 1 Page 1 lines 8-9 |
| Setting and location | 5 | State relevant aspects of the system(s) in which the decision(s) need(s) to be made. | Supplementary material 1 Page 1 lines 3-4 |
| Study perspective | 6 | Describe the perspective of the study and relate this to the costs being evaluated. | Supplementary material 1 Page 1 lines 5-7 |
| Comparators | 7 | Describe the interventions or strategies being compared and state why they were chosen. | Supplementary material 1 Page 1 lines 9-15 |
| Time horizon | 8 | State the time horizon(s) over which costs and consequences are being evaluated and say why appropriate. | Supplementary material 1 Page 2 lines 16-17 |
| Discount rate | 9 | Report the choice of discount rate(s) used for costs and outcomes and say why appropriate. | Supplementary material 1 Page 1 line 18 |
| Choice of health outcomes | 10 | Describe what outcomes were used as the measure(s) of benefit in the evaluation and their relevance for the type of analysis performed. | Supplementary material 1 Page 1 line 3 |
| Measurement of effectiveness | 11a | *Single study-based estimates:*Describe fully the design features of the single effectiveness study and why the single study was a sufficient source of clinical effectiveness data. | N/A |
|  | 11b | *Synthesis-based estimates:*Describe fully the methods used for identification of included studies and synthesis of clinical effectiveness data. | Supplementary material 1 Pages 6-8 |
| Measurement and valuation of preference based outcomes | 12 | If applicable, describe the population and methods used to elicit preferences for outcomes. | N/A |
| Estimating resources and costs | 13a | *Single study-based economic evaluation:*Describe approaches used to estimate resource use associated with the alternative interventions. Describe primary or secondary research methods for valuing each resource item in terms of its unit cost. Describe any adjustments made to approximate to opportunity costs. | N/A |
|  | 13b | *Model-based economic evaluation:*Describe approaches and data sources used to estimate resource use associated with model health states. Describe primary or secondary research methods for valuing each resource item in terms of its unit cost. Describe any adjustments made to approximate to opportunity costs. | Supplementary material 1 Pages 8-10 |
| Currency, price date, and conversion | 14 | Report the dates of the estimated resource quantities and unit costs. Describe methods for adjusting estimated unit costs to the year of reported costs if necessary. Describe methods for converting costs into a common currency base and the exchange rate. | Supplementary material 1 Page 8 lines 4-7 |
| Choice of model | 15 | Describe and give reasons for the specific type of decision-analytical model used. Providing a figure to show model structure is strongly recommended. | Supplementary material 1 Figure 1, Supplementary material 1 page 1 lines 22-24 |
| Assumptions | 16 | Describe all structural or other assumptions underpinning the decision-analytical model. | Supplementary material 1 Pages 3-10 |
| Analytical methods | 17 | Describe all analytical methods supporting the evaluation. This could include methods for dealing with skewed, missing, or censored data; extrapolation methods; methods for pooling data; approaches to validate or make adjustments (such as half cycle corrections) to a model; and methods for handling population heterogeneity and uncertainty. | Supplementary material 1 Pages 1-11 |
| **Results** | | | |
| Study parameters | 18 | Report the values, ranges, references, and, if used, probability distributions for all parameters. Report reasons or sources for distributions used to represent uncertainty where appropriate. Providing a table to show the input values is strongly recommended. | Supplementary material 1 table 1 |
| Incremental costs and outcomes | 19 | For each intervention, report mean values for the main categories of estimated costs and outcomes of interest, as well as mean differences between the comparator groups. If applicable, report incremental cost-effectiveness ratios. | Supplementary material 2 |
| Characterising uncertainty | 20a | *Single study-based economic evaluation:*Describe the effects of sampling uncertainty for the estimated incremental cost and incremental effectiveness parameters, together with the impact of methodological assumptions (such as discount rate, study perspective). | N/A |
|  | 20b | *Model-based economic evaluation:*Describe the effects on the results of uncertainty for all input parameters, and uncertainty related to the structure of the model and assumptions. | Supplementary material 2, Figure 1 |
| Characterising heterogeneity | 21 | If applicable, report differences in costs, outcomes, or cost-effectiveness that can be explained by variations between subgroups of patients with different baseline characteristics or other observed variability in effects that are not reducible by more information. | N/A |
| **Discussion** | | | |
| Study findings, limitations, generalisability, and current knowledge | 22 | Summarise key study findings and describe how they support the conclusions reached. Discuss limitations and the generalisability of the findings and how the findings fit with current knowledge. | Main text page 2 lines 7-18, limited by the type of article |
| **Other** | | | |
| Source of funding | 23 | Describe how the study was funded and the role of the funder in the identification, design, conduct, and reporting of the analysis. Describe other non-monetary sources of support. | Supplementary material 1 page 11 line 24 |
| Conflicts of interest | 24 | Describe any potential for conflict of interest of study contributors in accordance with journal policy. In the absence of a journal policy, we recommend authors comply with International Committee of Medical Journal Editors recommendations. | Supplementary material 1 page 11 line 25 |

# 2. Results

Supplementary material 2 Table 1. Results of the base case scenario.

|  | Costs per patient (£) | QALY per patient | Incremental costs (£) | Incremental QALYs | ICER (£/QALY) |
| --- | --- | --- | --- | --- | --- |
| Current practice | 321 490.94 | 6.561 | - | - | - |
| Liver Assist™ | 330 247.37 | 6.604 | 8 756.42 | 0.043 | 204 059.25 |
| Metra™ | 334 759.84 | 6.608 | 4 512.47 | 0.004 | 1 089 783.06 |

* SCS dominates as the intervention offers fewer QALY

Supplementary material 2 Table 2. Results of the one-way sensitivity analysis. All variables were analysed and variables that change the ICER by more than 3% are reported. All incremental costs and benefits compare the intervention to Static Cold Storage (SCS).

|  |  | Liver Assist ™ | | |  | Metra™ | |  |  |
| --- | --- | --- | --- | --- | --- | --- | --- | --- | --- |
| Parameter | Data source | Parameter change high | Parameter change low | % Change of ICER high | % Change of ICER low | Parameter change high | Parameter change low | % Change of ICER high | % Change of ICER low |
| RR of EAD | Jia et al. ^1^ (95% CI) | 80% | -45% | 239.72 | -23.48 | 63.6% | -40.9% | 290.85 | -20.38 |
| % of organ utilisation | Varied by % | 10% | -10% | -54.16 | -108.98* | 10.0% | -10.0% | -58.53 | -126.48* |
| Transition probability of developing EAD | Varied by % | 10% | -10% | -7.03 | 8.57 | 10.0% | -10.0% | -7.57 | 9.23 |
| Device Cost | Varied by % | 10% | -10% | 7.88 | -7.88 | 10.0% | -10.0% | 8.46 | -8.46 |
| Utility "AN" | Ratcliffe et al.^2^ (95% CI) | 4% | -4% | -4.74 | 5.23 | 3.9% | -3.9% | -4.74 | 5.23 |
| Relative risk PNF | Jia et al. ^1^ (95% CI) | 333% | -77% | 6.84 | -1.42 | 333.3% | -76.7% | 6.64 | -1.39 |
| Discount rate | NICE ^3^ | 1.5% | 0% | -8.32 | -13.97 | 1.5% | 0.0% | -9.07 | -15.23 |
| Alternate data for long term outcomes | Lee et al. ^4^ | 1 year outcomes | 5 year outcomes | 80.90 | 180.88 | 1 year outcomes | 5 year outcomes | 87.27 | 195.22 |
| Cost of the space for machine perfusion | Ang et al. ^5^, Varied by % | Theatre space | Perfusion room cost -10% | 1 561.31 | -0.26 | Theatre space | Perfusion room cost -10% | 224.44 | -0.17 |

# Bibliography

1 Jia J, Zheng S. A Systematic Review and Meta-Analysis of Machine Perfusion vs . Static Cold Storage of Liver Allografts on Liver Transplantation Outcomes : The Future Direction of Graft Preservation. 2020; **7**.

2 Ratcliffe J, Longworth L, Young T, Bryan S, Burroughs A, Buxton M. Assessing health-related quality of life pre- and post-liver transplantation: A prospective multicenter study. *Liver Transplant*. 2002; **8**: 263–270.

3 National Institute for Health and Care Excellence (NICE). The reference case | Guide to the methods of technology appraisal 2013 | Guidance | NICE [Internet]. [cited 2020 Aug 16]. Available from: https://www.nice.org.uk/process/pmg9/chapter/the-reference-case

4 Lee DD, Croome KP, Shalev JA, Musto KR, Sharma M, Keaveny AP, *et al.* Early allograft dysfunction after liver transplantation: An intermediate outcome measure for targeted improvements. *Ann Hepatol* [Internet]. Elsevier; 2016; **15**: 53–60. Available from: http://dx.doi.org/10.5604/16652681.1184212

5 Ang WW, Sabharwal S, Johannsson H, Bhattacharya R, Gupte CM. The cost of trauma operating theatre inefficiency. *Ann Med Surg*. Elsevier Ltd; 2016 May 1; **7**: 24–29.

# 3. Cochrane Risk of Bias tool

Revised Cochrane risk-of-bias tool for randomized trials (RoB 2)

TEMPLATE FOR COMPLETION

Edited by Julian PT Higgins, Jelena Savović, Matthew J Page, Jonathan AC Sterne
on behalf of the RoB2 Development Group

**Version of 22 August 2019**

The development of the RoB 2 tool was supported by the MRC Network of Hubs for Trials Methodology Research (MR/L004933/2- N61), with the support of the host MRC ConDuCT-II Hub (Collaboration and innovation for Difficult and Complex randomised controlled Trials In Invasive procedures - MR/K025643/1), by MRC research grant MR/M025209/1, and by a grant from The Cochrane Collaboration.


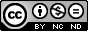


This work is licensed under a [Creative Commons Attribution-NonCommercial-NoDerivatives 4.0 International License](http://creativecommons.org/licenses/by-nc-nd/4.0/).

| **Study details**   \| **Reference** \| 1. Nasralla D, Coussios CC, Mergental H, Zeeshan Akhtar M, Butler AJ, Ceresa DL, et al. A randomized trial of normothermic preservation in liver transplantation. Nature [Internet]. 2018 [cited 2020 Apr 9]; Available from: https://doi.org/10.1038/s41586-018-0047-9 \| \| --- \| --- \|   **Study design**   \| X \| Individually-randomized parallel-group trial \| \| --- \| --- \| \| □ \| Cluster-randomized parallel-group trial \| \| □ \| Individually randomized cross-over (or other matched) trial \|   **For the purposes of this assessment, the interventions being compared are defined as**   \| Experimental: \| Normothermic machine perfusion \| Comparator: \| Static cold storage (current practice) \| \| --- \| --- \| --- \| --- \|  \| **Specify which outcome is being assessed for risk of bias** \| Discard rate \| \| --- \| --- \|  \| **Specify the numerical result being assessed.** In case of multiple alternative analyses being presented, specify the numeric result (e.g. RR = 1.52 (95% CI 0.83 to 2.77) and/or a reference (e.g. to a table, figure or paragraph) that uniquely defines the result being assessed. \| NMP: 16 (11.7%) of 121,  SCS: 32 (24.1%) of 101  Effect (95% CI): −12.4% (−21.4 to −3.3%)  P value: 0.008 \| \| --- \| --- \|   **Is the review team’s aim for this result…?**   \| X \| to assess the effect of *assignment to intervention* (the ‘intention-to-treat’ effect) \| \| --- \| --- \| \| □ \| to assess the effect of *adhering to intervention* (the ‘per-protocol’ effect) \|   **If the aim is to assess the effect of *adhering to intervention***, select the deviations from intended intervention that should be addressed (at least one must be checked):  □ occurrence of non-protocol interventions  □ failures in implementing the intervention that could have affected the outcome  □ non-adherence to their assigned intervention by trial participants  **Which of the following sources were obtained to help inform the risk-of-bias assessment? (tick as many as apply)**  x Journal article(s) with results of the trial  x Trial protocol  □ Statistical analysis plan (SAP)  x Non-commercial trial registry record (e.g. ClinicalTrials.gov record)  □ Company-owned trial registry record (e.g. GSK Clinical Study Register record)  □ “Grey literature” (e.g. unpublished thesis)  □ Conference abstract(s) about the trial  □ Regulatory document (e.g. Clinical Study Report, Drug Approval Package)  □ Research ethics application  □ Grant database summary (e.g. NIH RePORTER or Research Councils UK Gateway to Research)  □ Personal communication with trialist  □ Personal communication with the sponsor |
| --- | --- | --- | --- | --- | --- | --- | --- | --- | --- | --- | --- | --- | --- | --- | --- | --- | --- | --- | --- | --- |

Risk of bias assessment

Responses underlined in green are potential markers for low risk of bias, and responses in red are potential markers for a risk of bias. Where questions relate only to sign posts to other questions, no formatting is used.

**Domain 1: Risk of bias arising from the randomization process**

| **Signalling questions** | **Comments** | **Response options** |
| --- | --- | --- |
| **1.1 Was the allocation sequence random?** | “Using an online randomization tool, livers were assigned to NMP or SCS with  1:1 allocation ratio as per a computer-generated randomization schedule, using variable block size, stratified by transplant centre and donor type (DBD/DCD).” (Journal article)  “Once an eligible donor organ was allocated to a consented recipient and the availability of the NMP device and team was confirmed, the liver was randomized.” (Journal Article) | Y / PY / PN / N / NI  Y |
| **1.2 Was the allocation sequence concealed until participants were enrolled and assigned to interventions?** |  | Y / PY / PN / N / NI  Y |
| **1.3 Did baseline differences between intervention groups suggest a problem with the randomization process?** | See Table 1 (Donor) and Table 2 (Recipient) (Journal Article) | Y / PY / PN / N / NI  N |
| **Risk-of-bias judgement** |  | Low / High / Some concerns  Low |
| Optional: What is the predicted direction of bias arising from the randomization process? |  | NA / Favours experimental / Favours comparator / Towards null /Away from null / Unpredictable |

Domain 2: Risk of bias due to deviations from the intended interventions (*effect of assignment to intervention*)

| **Signalling questions** | **Comments** | **Response options** |
| --- | --- | --- |
| **2.1. Were participants aware of their assigned intervention during the trial?** | “Study design: Multicentre non-blinded randomised controlled trial” (Registry)  “This was an open label study.” (Journal Article)  “Whilst it is not possible to blind the local investigators to the method of organ preservation, outcome assessors will be blinded where possible. This includes the histopathologist interpreting the biopsy specimens as well as the radiologist interpreting the 6-month MRCP images.” (Trial protocol) | Y / PY / PN / N / NI  Y |
| **2.2. Were carers and people delivering the interventions aware of participants' assigned intervention during the trial?** |  | Y / PY / PN / N / NI  Y |
| **2.3. If Y/PY/NI to 2.1 or 2.2: Were there deviations from the intended intervention that arose because of the trial context?** | See Figure 2 (Journal article), livers were randomised, then excluded if for example the DCD did not proceed, then either discarded or transplanted. No criteria for discard described in the Journal article, trial protocol or registry. Reasons for discarding NMP livers provided in Extended data table 1 (Journal article) but no reasons for discard of SCS livers provided. The decision to discard an organ was a clinical decision and may have been subject to conscious or unconscious bias. | NA / Y / PY / PN / N / NI  PY |
| **2.4 If Y/PY to 2.3: Were these deviations likely to have affected the outcome?** | Investigators consciously or unconsciously could have made clinical decisions in accordance with the trial hypothesis, as the method of organ preservation was known to them. This would lead to a decision to discard more organs from the control group than from the intervention group. | NA / Y / PY / PN / N / NI  PY |
| **2.5. If Y/PY/NI to 2.4: Were these deviations from intended intervention balanced between groups?** | NMP - Successfully transplanted n = 121 Discarded n = 16  SCS - Successfully transplanted n = 101 Discarded n = 32  (Journal article Figure 2) | NA / Y / PY / PN / N / NI  N |
| **2.6 Was an appropriate analysis used to estimate the effect of assignment to intervention?** | “Results are reported as a modified intention-to-treat analysis. A per-protocol  sensitivity analysis was also performed excluding livers that received machine perfusion outside the protocol specified range (4–24 h) and comparing the groups according to the treatment actually received. Livers randomized but not retrieved were excluded from the analysis” (Journal Article) | Y / PY / PN / N / NI  Y |
| **2.7 If N/PN/NI to 2.6: Was there potential for a substantial impact (on the result) of the failure to analyse participants in the group to which they were randomized?** |  | NA / Y / PY / PN / N / NI  NA |
| **Risk-of-bias judgement** |  | Low / High / Some concerns  High |
| Optional: What is the predicted direction of bias due to deviations from intended interventions? |  | NA / Favours experimental / Favours comparator / Towards null /Away from null / Unpredictable |

Domain 2: Risk of bias due to deviations from the intended interventions (*effect of adhering to intervention*)

| **Signalling questions** | **Comments** | **Response options** |
| --- | --- | --- |
| **2.1. Were participants aware of their assigned intervention during the trial?** |  | Y / PY / PN / N / NI |
| **2.2. Were carers and people delivering the interventions aware of participants' assigned intervention during the trial?** |  | Y / PY / PN / N / NI |
| **2.3. [If applicable:] If Y/PY/NI to 2.1 or 2.2: Were important non-protocol interventions balanced across intervention groups?** |  | NA / Y / PY / PN / N / NI |
| **2.4. [If applicable:] Were there failures in implementing the intervention that could have affected the outcome?** |  | NA / Y / PY / PN / N / NI |
| **2.5. [If applicable:] Was there non-adherence to the assigned intervention regimen that could have affected participants’ outcomes?** |  | NA / Y / PY / PN / N / NI |
| **2.6. If N/PN/NI to 2.3, or Y/PY/NI to 2.4 or 2.5: Was an appropriate analysis used to estimate the effect of adhering to the intervention?** |  | NA / Y / PY / PN / N / NI |
| **Risk-of-bias judgement** |  | Low / High / Some concerns |
| Optional: What is the predicted direction of bias due to deviations from intended interventions? |  | NA / Favours experimental / Favours comparator / Towards null /Away from null / Unpredictable |

Domain 3: Missing outcome data

| **Signalling questions** | **Comments** | **Response options** |
| --- | --- | --- |
| **3.1 Were data for this outcome available for all, or nearly all, participants randomized?** | Yes, see figure 2 (Journal article) | Y / PY / PN / N / NI  Y |
| **3.2 If N/PN/NI to 3.1: Is there evidence that the result was not biased by missing outcome data?** |  | NA / Y / PY / PN / N |
| **3.3 If N/PN to 3.2: Could missingness in the outcome depend on its true value?** |  | NA / Y / PY / PN / N / NI |
| **3.4 If Y/PY/NI to 3.3: Is it likely that missingness in the outcome depended on its true value?** |  | NA / Y / PY / PN / N / NI |
| **Risk-of-bias judgement** |  | Low / High / Some concerns  Low |
| Optional: What is the predicted direction of bias due to missing outcome data? |  | NA / Favours experimental / Favours comparator / Towards null /Away from null / Unpredictable |

Domain 4: Risk of bias in measurement of the outcome

| **Signalling questions** | **Comments** | **Response options** |
| --- | --- | --- |
| **4.1 Was the method of measuring the outcome inappropriate?** | No criteria for discarding organs described in the Journal article, trial protocol or registry. | Y / PY / PN / N / NI  NI |
| **4.2 Could measurement or ascertainment of the outcome have differed between intervention groups?** | No criteria for discard described in the Journal article, trial protocol or registry. Reasons for discarding NMP livers provided in Extended data table 1 (Journal article) but no reasons for discard of SCS livers provided. | Y / PY / PN / N / NI  NI/PY |
| **4.3 If N/PN/NI to 4.1 and 4.2: Were outcome assessors aware of the intervention received by study participants?** | Yes, open label study | NA / Y / PY / PN / N / NI  Y |
| **4.4 If Y/PY/NI to 4.3: Could assessment of the outcome have been influenced by knowledge of intervention received?** | The decision to discard an organ or proceed to transplantation is clinical as no objective criteria have been published. It was therefore likely to be subject to conscious or unconscious bias. Investigators would likely have made decisions in accordance with the trial hypothesis as the method of organ preservation was known to them. | NA / Y / PY / PN / N / NI  Y |
| **4.5 If Y/PY/NI to 4.4:** **Is it likely that assessment of the outcome was influenced by knowledge of intervention received?** |  | NA / Y / PY / PN / N / NI  Y |
| **Risk-of-bias judgement** |  | Low / High / Some concerns  HIgh |
| Optional: What is the predicted direction of bias in measurement of the outcome? |  | NA / Favours experimental / Favours comparator / Towards null /Away from null / Unpredictable |

Domain 5: Risk of bias in selection of the reported result

| **Signalling questions** | **Comments** | **Response options** |
| --- | --- | --- |
| **5.1 Were the data that produced this result analysed in accordance with a pre-specified analysis plan that was finalized before unblinded outcome data were available for analysis?** | Statistical analysis on p.50 of the Trial protocol. “Full details of the proposed statistical analysis will be outlined in a separate document (the Statistical Analysis Plan; SAP).” (Trial protocol) | Y / PY / PN / N / NI  Y |
| **Is the numerical result being assessed likely to have been selected, on the basis of the results, from...** |  |  |
| **5.2. ... multiple eligible outcome measurements (e.g. scales, definitions, time points) within the outcome domain?** | No | Y / PY / PN / N / NI  N |
| **5.3 ... multiple eligible analyses of the data?** | No | Y / PY / PN / N / NI  N |
| **Risk-of-bias judgement** |  | Low / High / Some concerns  Low |
| Optional: What is the predicted direction of bias due to selection of the reported result? |  | NA / Favours experimental / Favours comparator / Towards null /Away from null / Unpredictable |

Overall risk of bias

| **Risk-of-bias judgement** |  | Low / High / Some concerns  High |
| --- | --- | --- |
| Optional: What is the overall predicted direction of bias for this outcome? |  | NA / Favours experimental / Favours comparator / Towards null /Away from null / Unpredictable |


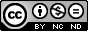


This work is licensed under a [Creative Commons Attribution-NonCommercial-NoDerivatives 4.0 International License](http://creativecommons.org/licenses/by-nc-nd/4.0/)
